# Perspectives and potential applications of endophytic microorganisms in cultivation of medicinal and aromatic plants

**DOI:** 10.3389/fpls.2022.985429

**Published:** 2022-09-29

**Authors:** Arpita Tripathi, Praveen Pandey, Shakti Nath Tripathi, Alok Kalra

**Affiliations:** ^1^ Microbial Technology Department, CSIR-Central Institute of Medicinal and Aromatic Plants, Lucknow, India; ^2^ Academy of Scientific and Innovative Research (AcSIR), Ghaziabad, India; ^3^ Faculty of Education, Teerthanker Mahaveer University, Moradabad, India; ^4^ Division of Plant Breeding and Genetic Resource Conservation, CSIR-Central Institute of Medicinal and Aromatic Plants, Lucknow, India; ^5^ Department of Botany, Nehru Gram Bharati Deemed to be University, Prayagraj, India

**Keywords:** plant-microbe interaction, plant growth promotion, secondary metabolites, stress tolerance, medicinal plants, endophytic microorganisms

## Abstract

Ensuring food and nutritional security, it is crucial to use chemicals in agriculture to boost yields and protect the crops against biotic and abiotic perturbations. Conversely, excessive use of chemicals has led to many deleterious effects on the environment like pollution of soil, water, and air; loss of soil fertility; and development of pest resistance, and is now posing serious threats to biodiversity. Therefore, farming systems need to be upgraded towards the use of biological agents to retain agricultural and environmental sustainability. Plants exhibit a huge and varied niche for endophytic microorganisms inside the *planta*, resulting in a closer association between them. Endophytic microorganisms play pivotal roles in plant physiological and morphological characteristics, including growth promotion, survival, and fitness. Their mechanism of action includes both direct and indirect, such as mineral phosphate solubilization, fixating nitrogen, synthesis of auxins, production of siderophore, and various phytohormones. Medicinal and aromatic plants (MAPs) hold a crucial position worldwide for their valued essential oils and several phytopharmaceutically important bioactive compounds since ancient times; conversely, owing to the high demand for natural products, commercial cultivation of MAPs is on the upswing. Furthermore, the vulnerability to various pests and diseases enforces noteworthy production restraints that affect both crop yield and quality. Efforts have been made towards enhancing yields of plant crude drugs by improving crop varieties, cell cultures, transgenic plants, etc., but these are highly cost-demanding and time-consuming measures. Thus, it is essential to evolve efficient, eco-friendly, cost-effective simpler approaches for improvement in the yield and health of the plants. Harnessing endophytic microorganisms as biostimulants can be an effective and alternative step. This review summarizes the concept of endophytes, their multidimensional interaction inside the host plant, and the salient benefits associated with endophytic microorganisms in MAPs.

## Introduction

Plants perform a range of fundamental functions in helping all distinct forms of living beings and release chemical signals to communicate with them. The roots give anchorage to the plant in soil and assist in the acquisition of water and nutrients, as well as produce chemical compounds that induce various types of interactions. This constitutes mutualism with the microorganisms that include fungi in mycorrhizal associations, endophytes, and plant growth-promoting rhizobacteria (PGPRs) as well as parasitism with pathogenic microorganisms, other plants, and herbivores (Badri et al., 2009). The plant roots discharge tremendous quantities of chemical compounds to fight pathogens and attract advantageous microorganisms (Badri and Vivanco, 2009). Such plant–microbe interactions occur at different trophic levels within a sophisticated arrangement of communities (Yan et al., 2019). Approximately 470 million years ago, the evolution of aquatic plants into terrestrial organisms was made possible by cooperating with soil microbes, and many of those microbe–plant interactions still persist (Bhattacharya and Medlin, 1998; Palmer et al., 2004; Ni et al., 2012; Williams et al., 2013). Some plant–microbe interactions are commensalism, where no harm is done to the plant, but the microbe gains some advantage. Several distinct interactions are advantageous to both partners (e.g., they are called mutualistic). Lastly, another group of microorganisms are pathogens and parasites to their host plants. In all the incidents, the microbe and the plant have established the ability to communicate. The microorganism recognizes and responds to the chemical signaling molecules produced by the plants. This usually results in the discharge of microbial compounds that are in turn identified by the plant, thereby generating a two-way “communication” that uses a molecular lexicon. Once a plant–microbe relationship begins, microbes and plants continue to observe their partner’s physiology and coordinate their activities accordingly.

Medicinal and aromatic plants (MAPs) hold a crucial position within people’s healthcare systems throughout the world. Until the arrival of advanced medicines, an oversized population in emerging nations has traditionally relied upon the products obtained from plants, particularly from forests. Numerous medicinal and aromatic crops are being exploited for economic uses. Approximately 12.5% of the more than 422,000 plant species have been universally documented for medicinal properties; however, only a couple of hundreds are known to be in cultivation (Schippmann et al., 2002). There is a need to grow MAPs to maintain their steady supply and conservation amidst decreasing stocks from natural sources and rising global interest. Apart from increasing farmers’ income, MAPs cultivation also acts as insurance crops against the climate extremes. Its cultivation for essential oils and several phytopharmaceutically important compounds is an age-old aspect of agriculture; conversely, owing to the high demand for natural products, commercial cultivation of MAPs is on the upswing. Furthermore, the vulnerability to various biotic and abiotic perturbations (phytopathogens, drought, water-logging, salinity, temperature, etc.) enforces noteworthy production constraints to diminish both yield and overall quality of the crops. Until the arrival of today’s medicine, a large part of the population in developing nations traditionally depended on the products obtained from plants, particularly from forests, for administering human and livestock ailments. Some efforts have been made towards enhancing yields of plant crude drugs by improving crop varieties, cell cultures, transgenic plants, etc., but these are highly cost-demanding and time-consuming measures. Thus, it is vital to evolve efficient, eco-friendly, cost-effective simpler approaches for improvement in yield and health of the plants.

Plant growth-promoting endophytic microorganisms inhabit and proliferate inside the plants without any distinct symptoms of any diseases *in-planta* (Bacon et al., 2002), and their mechanism includes both direct and indirect actions such as mineral phosphate solubilization (Verma et al., 2001), fixating nitrogen (Alishahi et al., 2020), synthesis of auxins (Patten and Glick, 2002), production of siderophore (Lodewyckx et al., 2002), and various phytohormones (Costacurta and Vanderleyden, 1995). As many abiotic stresses induce multiple physiological disturbances, which include stomatal closure and stunted plant growth, this ultimately results in lesser crop yield. It has been well reported that ACC (1-aminocyclopropane-1-carboxylic acid) deaminase-containing microbes lower the effect of stress induced by higher ethylene levels of the host plant (Penrose et al., 2001; Sessitsch et al., 2005; Glick, 2005; Sun et al., 2009; Rashid et al., 2012). Therefore, microbial treatments can protect plants from the damaging effects of environmental perturbations (Ali et al., 2014). Changes in climatic conditions such as rainfall, ambient CO_2_, and varying temperatures affect agriculture through countless constraints resulting in either low yields or sometimes death of the plants. This review reveals the role of endophytes in improving agricultural sustainability, which can serve as a valuable approach toward green cultivation of MAPs and cost-effective drug production.

## Endophytes: Microbial entities for plant fitness

Endophytes, the Greek word having “endon” (within) and “phyton” (plant), was coined by De Bary (1879) for “any organism occurring within plant tissues”. Bacon and White (2000) have defined endophyte in the broadest and widely accepted manner that states that endophytes include those microbes colonizing living plant internal tissues and not causing any instant, obvious ill effects. Thus, most precisely, endophytes refer to microorganisms (fungi, bacteria, actinomycetes, etc.) that spend at least a part of their life cycle establishing a relationship with a plant that remains asymptomatic (Vanessa and Christopher, 2004). Microorganisms that require living cells to grow and complete their life cycle are known as “obligate” while the others that mainly thrive on the outside of the plant tissues are termed as “epiphytes” and sometimes may enter the plant endosphere, called “opportunistic” (Hardoim et al., 2008). In this interaction, both plant and endophytic microbes live together, providing profound benefits to each other (Ting et al., 2009). These endophytes are often rhizospheric in nature, and preferable sites for their attachment and subsequent entry into the host plant could be apical root zone with thin-walled surface root layers and basal root zones with small cracks (Gagné, 1987). They proliferate in the entire host plant (Hallmann et al., 1997a), and reside within the cells, vascular tissues, or intercellular spaces (Patriquin and Dobereiner, 1978; Jacobs et al., 1985; Bell et al., 1995). Endophytic microbes could also enter through the stomata and vertically transmit from parent to offspring *via* seeds while roots have maximum colonization through epidermis formed by lateral root emergence (Roos and Hattingh, 1983; Agrawal and Shende, 1987). Which community of microbes are friends and which are foes? It is decided by the immune system of the plant itself (Zeilinger et al., 2016). The “balanced antagonism” with asymptomatic colonization between the host plant and endophytic microbes clearly shows that endophytes can live within the plant without activating any host defense mechanism and improves its self-sustenance through the production of the plant-like substances (Schulz et al., 1999; Schulz and Boyle, 2005).

The study of plant–microbe interactions helps us acknowledge natural events that influence our daily lives and could benefit befalling in sustainable resources, a smaller influence on the atmosphere and surroundings, and control of environmental pollution. The benefits of using these interactions for biotechnological applications are huge. The utilization of the pre-existing plant–microbe interactions for the promotion of growth of the plant and biocontrol diminishes the use of unnatural synthetic pesticides and fertilizers, resulting in lowering input costs and, more importantly, reducing the influence of chemical nutrients and pesticides on existing useful fiora and fauna (Boddey et al., 2003; Elmer and Reglinski, 2006; Whipps and Gerhardson, 2007). The production of beneficial compounds of industrial and pharmaceutical importance through plant–microbe symbiosis reduces the requirement to supply expensive catalysts and precursors and is energy-saving (Wu et al., 2007; Del Giudice et al., 2008). Remediation by traditional methods is costly and laborious; however, plant–microbe remediation approaches are incredibly efficient and less interfering (Anderson et al., 1993). The carbon sequestration by the plant–rhizosphere methods is probably a sustainable approach for reducing atmospheric carbon (Wu et al., 2009).

As the name implies, endophytic microorganisms live within a plant in the intercellular spaces of various plant parts such as stems, roots, petioles, leaves, etc., without imposing any apparent symptoms of disease or ill health. The symbiotic relationship of the host and its endophytes has been studied in detail and is explained as the plant partner protecting and feeding the endophytes, which, “in return”, produce certain substances with bioactive capabilities (antiviral, plant growth promotion, antibacterial, antifungal, insecticidal, etc.) to augment the growth and competitiveness of the former under natural conditions. These endophytic entities defend their host plants from pathogens by secreting bioactive secondary metabolites under unfavorable environments (Azevedo et al., 2000; Strobel, 2003). The endophytic organisms are now recognized as a vital part of biodiversity, the allocation of endophytic microfiora varies with the difference according to its host. Zhang et al. (2006a) suggested that almost all of the vascular plants are known to harbor endophytic entities, especially those with medicinal values that are thought to be related to the formation of therapeutic products. Endophytic microbes are not entirely explored yet, but several investigations present them as an enormous therapeutic compound source. Worldwide, about 300,000 plants grown in an unexplored region are a host of at least one or more endophytic microbes (Araújo et al., 2001). Therefore, functionally diverse endophytes’ occurrence offers a key role in ecosystems at the most plentiful biodiversity (Strobel, 2003). Survival of the endophytic microbes inside the host cell can be for a long duration. The ability of these microbes to produce bioactive secondary metabolites makes them interesting candidates to be studied and exploited in biotechnological aspects (Casella et al., 2013) to add on to the existing wealth of secondary metabolites. Such interest has been reflected in a number of recent reviews showcasing the secondary metabolite-producing abilities of these wonder microbes (Müller, 2015). The growth and yield of several medicinal plants have been reported to be enhanced by many endophytes (Barnawal et al., 2012; Barnawal et al., 2014), especially by gene expression modulation regulated in important secondary metabolites’ biosynthesis. They have also been reported to impart tolerance to the plants against a range of biotic and abiotic stresses (Pandey et al., 2016a; Pandey et al., 2016b; Lata et al., 2018).

Chemical fertilizers’ indiscriminate use for boosting the productivity of crops is drastically destroying soil and environmental health (Tilman, 1998). Even then, the use of fertilizers is likely to increase further in agriculture to feed the ever-growing huge population (Vitousek et al., 1997; Frink et al., 1999). In the current scenario of global climate change, sustainable agriculture production proves to be a significant challenge. Some approaches such as the integration of microbes associated with plants in agriculture enhance the growth of the plant through different modes and alleviate several biotic and abiotic perturbations (Tanaka et al., 2005; Vega et al., 2008; Lata et al., 2018), and may serve as rescue practices under such situations.

## Colonization of the endophytic microorganisms in plants

**Figure 1:**
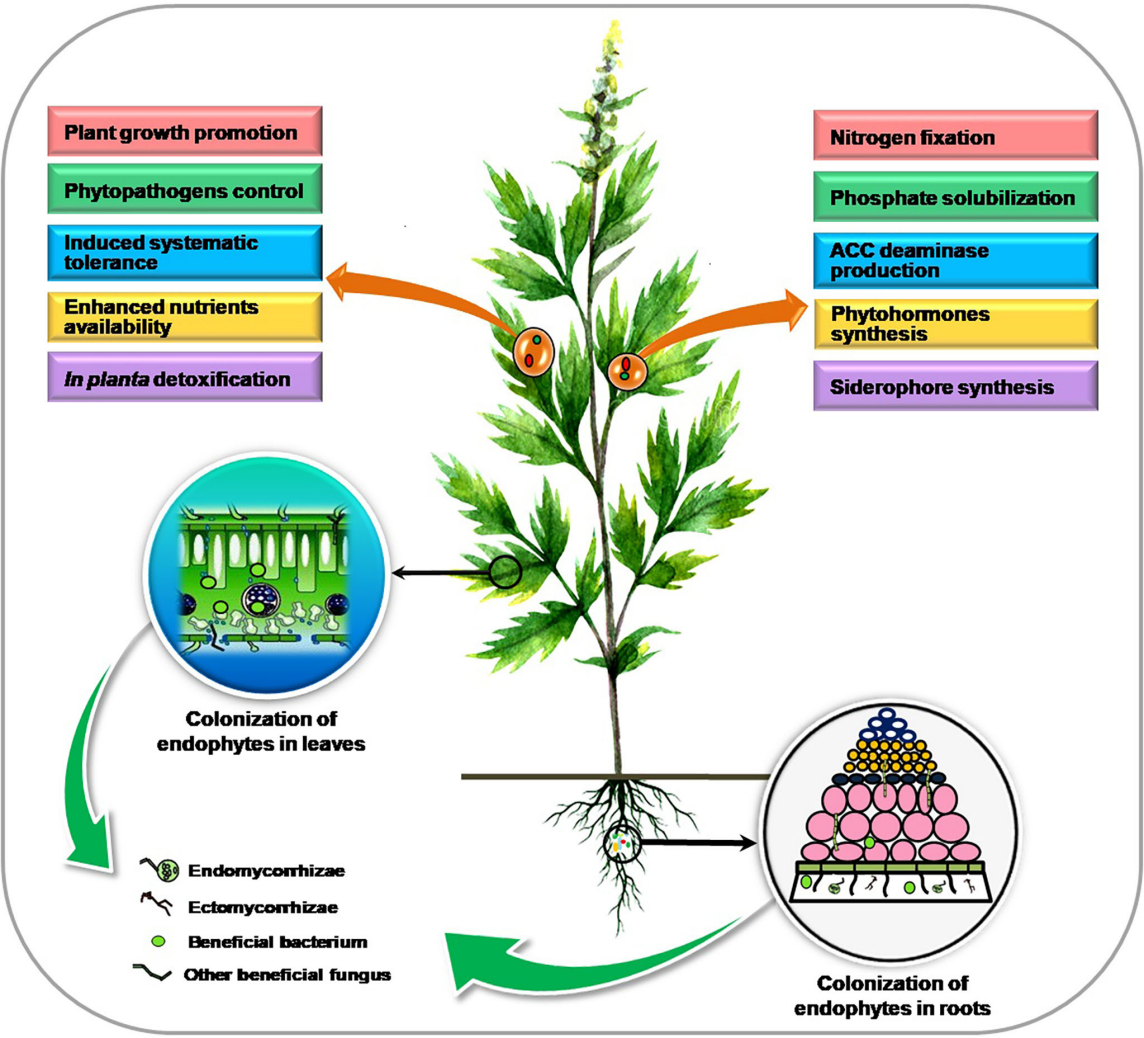
Colonization and benefits of endophytic microorganisms in plants.

Plants exhibit a huge and varied niche for microorganisms inside the *planta* resulting in a closer association between them. Endophytes inhabit the internal plant tissue comprising different bacterial and fungal species that collectively form the “plant endomicrobiome” and can trigger various physiological responses in the plant. Colonization and benefits associated with endophytic microorganisms in plants are presented in **Figure 1**.

## Colonization of the bacterial endophytes

Bacterial endophytes, sometimes considered a part of the population of rhizospheric microbes (Marquez-Santacruz et al., 2010), are present in various tissues such as the stem, root, leaf, tuber, and fruit, of different agricultural, horticultural, and forest species. As the endophytic bacteria are directly in contact with the plant tissues, they have the benefit of greater advantage than rhizospheric bacteria from the plants and offer more benefits to the plant other than bacteria in the rhizospheric region and outside the plants (Araujo et al., 2002). The wide-ranging and recurrent occurrence of such endophytic bacterial populations indicates that healthy plants recruit more endophytic populations (Hardoim et al., 2015) and provide these organisms with a huge and somewhat uncharted ecological niche.

The colonization of host-associated microbes occurs horizontally from the surroundings and might be vertical *via* parent to the progenies (Santoyo et al., 2016). Endophytic bacteria have several ways to enter inside the host plant tissues. Except for those microbes that are already established inside the seeds of the plant (Truyens et al., 2014), the most frequent point of entry of endophytic bacterial endophytes inside the host plant is *via* the cracks on the roots and wounded root tissues formed as a result of growth and development of the plant (Sprent and De Faria, 1989; Sørensen and Sessitsch, 2006). This allows leakage of plant metabolites, which attract more bacteria towards it (Hallmann et al., 1997a). Other points of entry of endophytes could be lenticels present in root and shoot periderm (Scott et al., 1996), radicles of germinating seeds, or lateral root hair cells. For example, Hallmann et al. (1997b) showed that *Enterobacter asburiae* JM22, an endophytic bacterium in cotton plants, produces enzymes capable of hydrolyzing cellulose in the cell wall, assisting the entry of the bacterium inside the host plant.

Autofluorescent proteins (AFP) could be an important method as well as a tool for the visualization of the biofilm to study plant–microbe interaction. These visualization techniques also include gene expression studies using GFP (green fluorescent proteins) in which the GFP gene is integrated into the chromosome of bacteria and a plasmid containing GFP cloned cells which are visualized by confocal or epifluorescence or laser scanning microscopy (Villacieros et al., 2003; Germaine et al., 2004). In the β-glucuronidase (GUS) reporter system, staining helps in the visualization of bacterial movement and gene expression in the rhizosphere and phyllosphere, and entrance and site of pathogens can be studied by IVET (*in vivo* expression technology) (Preston et al., 2001; Leveau and Lindow, 2001; Zhang et al., 2006a).

## Colonization of the fungal endophytes

For the recruitment of the endophytes, the host plant establishes symbiosis with large soil microbial diversity. Initially, attachment of the endophytic fungi might occur on the surface of roots and form structures called appressorium (Yedidia et al., 1999). After that, these attachments penetrate the outer root system and internally colonize the plant tissues (Viterbo and Chet, 2006; Nogueira-Lopez et al., 2018). Endophytic fungi mostly use two types of diffusion patterns; in the primary mechanism, fungi are vertically transmitted into progeny seed from maternal plants by which the offspring gets infected (Gagic et al., 2018). The endophytic fungi transmission among the host plants and the offspring is brought about under appropriate environmental conditions when infected seeds germinate and the endophytic fungi present inside the seed enter the seedlings after the germination of seeds (Hodgson et al., 2014).

Generally, endophytic fungi initiate from the nutrient-rich atmosphere of the rhizosphere, which also has insects and animal feeding processes, and air floating fungal spores (Rodriguez et al., 2009; Sasse et al., 2018). Many of the endophytic fungal microbes are transmitted through spores or hyphal fragments horizontally in aboveground tissues, by insects or herbivores (biotic) or rain or wind (abiotic dispersion agents) from plant to plant, thus establishing communication of fungal endophytes between several plant hosts (Wiewiora et al., 2015).

Microscopic observations of tomato roots on early colonization by the endophytic fungus *Trichoderma* illustrated no disturbance in cell integrity during this process (Chacón et al., 2007). However, in cucumber roots colonized by endophytes, various phenomena such as enhanced chitinase activity, necrosis of the penetration peg, and fluorescent products’ formation in the intercellular spaces were observed. This phenomenon might be due to copious extracellular enzyme production by endophytic fungi (Suryanarayanan et al., 2012).

## How endophytic microorganisms benefit the plants

**Figure 2:**
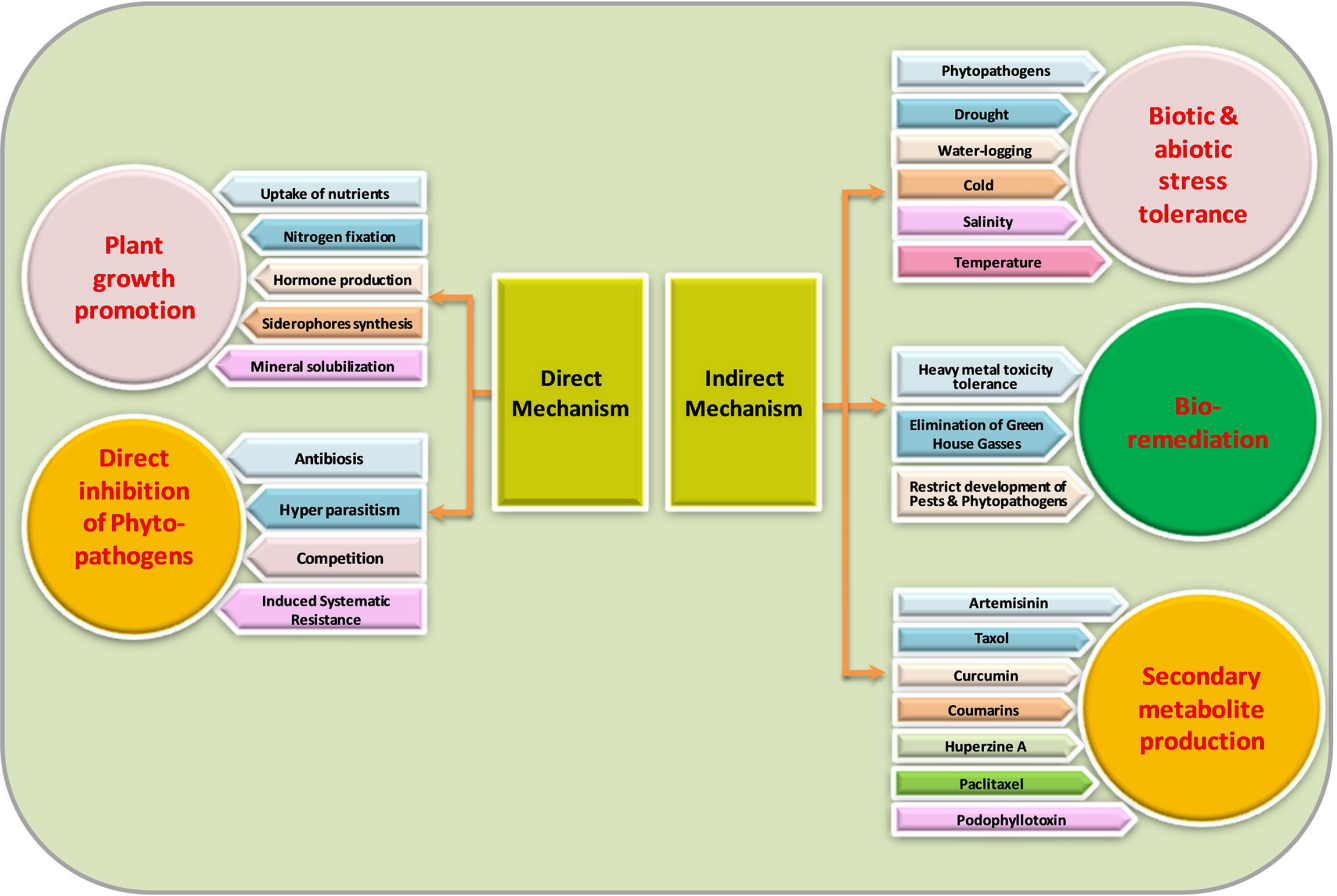
Mechanism of action of endophytic microorganisms.

Not much information exists about the mechanisms of endophyte-mediated plant growth enhancement. Endophytes can mediate plant growth improvement both directly and indirectly (**Figure 2**). As rhizospheric bacterium initiates its development into endophytes, it is supposed that endophytes can maintain their characteristics inside the host plant.

## Direct beneficial mechanisms

Endophytic microbes help host plants directly in many ways to promote the plant’s growth by improving the uptake of essential nutrients, which ultimately increases overall crop yield (Muthukumarasamy et al., 2002). A typical example is nitrogen fixation by particular endophytes in leguminous crops (Stacey et al., 2006). Additionally, several researchers have stated that endophytic bacteria effectively associate with non-leguminous crops to form a synergistic association intended for nitrogen fixation (Bhattacharjee et al., 2008; Saravanan et al., 2008; Mattos et al., 2008; Govindarajan et al., 2008; Oliveira et al., 2009). Plant growth enhancement may occur *via* many approaches that include plant hormone production, like gibberellins, IAA (indole-3-acetic acid), ethylene, and cytokinin regulation and activity. Many endophytes have shown to exhibit an activity of enzyme ACC (1-aminocyclopropane-1-carboxylate) deaminase to modulate physiology by reducing ethylene content (Hardoim et al., 2008; Barnawal et al., 2012) since ethylene in plant growth inhibitive hormone. In previous studies, *Burkholderia phytofirmans*, a plant growth-promoting endophytic strain, has been proven to enhance the yields of numerous crops (Lazarovits and Nowak, 1997; Sharma and Nowak, 1998; Sessitsch et al., 2005; Barka et al., 2006). Similarly, Mattos et al. (2008) showed that *Burkholderia kururiensis*, an endophytic bacterium, enhances IAA hormone production. *Penicillium citrinum* strain generated a large amount of physiologically active gibberellins than *Gibberlla fujikuroi* (wild type), which offers to generate a biologically active source of gibberellic acid (GA3) (Khan et al., 2008). Various studies showed that a variety of fungal endophytes promoted height, biomass, and tiller number in numerous crops (Spiering et al., 2006; Zhang et al., 2007; Lowe et al., 2008). *Stagonospora* spp., a seed-borne endophytic fungus, increased the yield of *Phragmites australis* (Cav.) Trin. ex Steud. (Ernst et al., 2003), and endophytic fungus promoted growth in peppermint (Mucciarelli et al., 2003). Some examples of endophytic microbes’ associated benefits in medicinal and aromatic plants are presented in **Table 1**.

**Table 1:** Benefits conferred by endophytic microorganisms in medicinal and aromatic plants.

Endophytic microorganisms	Host plant	Associated benefits	References
**Bacterial endophytes**			
*Pseudomonas putida BP25*	*Piper nigrum* (L.)	Inhibition of phytopathogens	Sheoran et al., 2015
*Bacillus licheniformi, B. subtilis, B. circulan, B. amyloliquefacien, B. licheniformi, Arthrobacter, Marmoricola* sp., *Acinetobacte, Microbacterium, Kocuria* sp., *Janibacter*	*Papaver somniferum* (L.)	Plant productivity and alkaloid biosynthesis	Pandey et al., 2016a
*Gordonea terrae*	*Avicennia marina* (Forssk.) Vierh.	Plant growth promotion	Soldan et al., 2019
*Pantoea*, *Pseudomonas*, *Enterobacter*	*Eleusine coracana* (L.)	Plant growth promotion	Misganaw et al., 2019
*Burkholderia phytofirmans strain* PsJN	*Vitis vinifera* (L.)	Plant growth promotion, enhancement of chilling resistance	Barka et al., 2006
*Bacillus subtilis LE24, Bacillus amyloliquefaciens LE109, Bacillus tequilensis PO80*	*Citrus* (L.)	Biocontrol of pathogens	Daungfu et al., 2019
*Enterobacter* sp. SA187		Plant growth promotion and salinity stress tolerance	de Zélicourt et al., 2018
*Burkholderia* sp.	*Helianthus annuus* (L.)	Calcium and phosphate solubilization	Ambrosini et al., 2012
**Fungal endophytes**			
*Mucor* sp.	*Arabidopsis arenosa* (L.) Lawalrée	Metal toxicity tolerance	Domka et al., 2019
*Colletotrichum tropicale*	*Theobroma cacao* (L.)	Tolerance to *Phytophthora*	Mejia et al., 2008
*Aspergillus fumigatus TS1, Fusarium proliferatum BRL1*	*Oxalis corniculata* (L.)	Plant growth promotion	Bilal et al., 2018
*Yarrowia lipolytica*	*Euphorbia milii* Des Moul.	Plant growth promotion and salinity stress tolerance	Jan et al., 2019
*Penicillium citrinum LWL4, Aspergillus terreus LWL5*	*Helianthus annuus* (L.)	Plant growth promotion, disease resistance	Waqas et al., 2015b
*Paecilomyces variotii, Penicillium purpurogenum*	*Caralluma acutangula* (Decne.) N.E.Br.	Plant growth promotion	Ali et al., 2019
*Sclerotium* sp.	*Atracty lancea* (Thunb.) DC.	Increases cell protection from desiccationin and leaf metabolic capability of host	Chen et al., 2008
*Epulorhiza* sp.	*Anoectochillus formosanus* Hayata	Enhances enzyme activities	Tang et al., 2008
*Epulorhiza* sp., *Mycena anoectochila*	*Anoectochilus roxburghii* (Wall.) Lindl.	Enhances enzyme activities	Yu and Guo, 2000; Chen et al., 2005
*Piriformospora indica*	*Cymbidium aloifolium* (L.) Sw.	Plant growth promotion and abiotic stress tolerance	Shah et al., 2019a
*Mycena orchdicola*	*Cymbidium sinense* Willd.	Secretes phytohormones	Zhang et al., 1999
*Fusarium* sp.	*Dendrobium moniliforme* (L.) Sw.	Plant growth promotion	Shah et al., 2019b
*Mycena dendrobii*	*Dendrobium candidum* Wall. ex Lindl.	Secretes phytohormones	Zhang et al., 1999
*Epulorhiza* sp., *Mycena* sp., *Sebacinales, Cantharellales*	*Dendrobium nobile* Lindl., *D. chrysanthum* Wall.	Enhances the nutrient absorption in plants, promoting the seed germination of host	Chen and Guo, 2005
*Aspergillus awamori* W11	*Withania somnifera* (L.)	Plant growth promotion	Mehmood et al., 2019
*Aspergillus terreus*, *Penicillium oxalicum, Sarocladium kiliense*		Biosynthesis of withanolide	Kushwaha et al., 2019a
*Aspergillus terreus, Penicillium oxalicum, Sarocladium kiliense*		Plant growth promotion, enhances withanolide content	Kushwaha et al., 2019b
*Mycena dendrobii, M. osmundicola, Mycena orchidicola, M. anoectochili*	*Gastrodia elata* Blume	Secretes phytohormones, promoting seed germination	Guo and Wang, 2001
*Epulorhiza* sp., *Fusarium* sp.	*Pecteilis susannae* (L.) Raf.	Enhances NPK absorption plants promoting the seed germination of host	Chutima et al., 2011
*Penicillium* sp., *Aspergillus* sp.	*Monochoria vaginalis* (Burm.f.) C.Presl ex Kunth	Secretes gibberellins	Ahmad et al., 2010
Dark septate endophytic fungi (DSEF)	*Pedicularis* (L.)	Increases their nutrient utilization efficiency	Li and Guan, 2007
*Ceratobasidium* sp.	*Rehmannia glutinosa* Steud.	Secretes IAA	Chen et al., 2011a
*Sebacina vermifera*	*Nicotiana attenuata* Steud.	Enhances absorption of nutrient and promote the growth and fitness by inhibiting ethylene signaling	Barazani et al., 2007
*Funneliformis mosseae, Rhizophagus intraradices,Claroideoglomus etunicatum*	*Sesbania sesban* (L.) Merr.	Secretes phytohormones	Abd Allah et al., 2015
*Chaetomium globosum, Aspergillus proliferans, Purpureocillium lilacinum*	*Papaver somniferum* (L.)	Enhances plant productivity and benzylisoquinoline alkaloid (BIA) biosynthesis	Pandey et al., 2016a
*Curvularia* sp., *Choanephora infundibulifera*	*Catharanthus roseus* (L.)	Terpenoid indole alkaloid biosynthesis	Pandey et al., 2016b

## Indirect beneficial mechanisms

Plants muddle through various unfavorable environmental conditions or abiotic stresses like cold, drought, hyper-salty situations, or pathogenesis. Endophytic microbes help the plant conquer such perturbations through some indirect mechanisms, which also promote the buildup of secondary metabolites (including drugs or important medicinal components) in plants.

The endophytic microbes support the host plants to defeat the before-mentioned stresses by some indirect mechanisms. They are also effective in bioremediation by various means, like they decrease heavy metal stress (Zhang et al., 2012), eliminating harmful greenhouse gases (Stępniewska and Kuźniar, 2013), and restricting the development of pests on plants (Azevedo et al., 2000). Endophytic microbes also support phytoremediation by reducing metal phytotoxicity. Simultaneously, for plants containing endophytes with requisite metabolic capabilities and degradation pathways for reducing phytotoxicity and magnifying degradation, the plant–endophyte relationships can be equipped to remediate wastelands and groundwater (Weyens et al., 2009). Rajkumar et al. (2009) reported that endophytic bacteria assist in promoting the extraction of heavy metals. Endophytes are also effective in the degradation of polyaromatic hydrocarbon (PAH). Radwan (2009) documented the phytoremediation of oily soils using rhizobacteria, which appears to be a cheap and environmentally friendly method of cleaning the environment.

## Tolerance to biotic stresses

Endophytic microorganisms have the ability to enhance plant resistance systems against pathogen infestation through antagonistic activity (Miller et al., 2002; Gunatilaka, 2006). Many studies showed that endophytes have a crucial role in regulating the gene expression of the host, modulating physiological responses and plant defense-related pathways (Van Bael et al., 2012; Estrada et al., 2013; Salam et al., 2017). Khare et al. (2016) illustrated that jasmonic acid and salicylic acid could greatly contribute to plant stress responses against phytopathogens. Ren and Dai (2012) described that inoculation with *Gilmaniella* sp. AL12 induces jasmonic acid defense responses against pathogenic fungi in *Atractylodes lancea* (Thunb.) DC. The gibberellins produced by endophytes enhance insect and phytopathogens’ resistance *via* salicylic and jasmonic acid pathways (Waqas et al., 2015a). An endophyte, *Fusarium solani*, elicits induced systemic resistance against a fungal pathogen, *Septoria lycopersici*, by stimulating gene expression linked to the pathogenesis (Kavroulakis et al., 2007). Foliar endophytic fungi, *Colletotrichum tropicale*, inoculated in *Theobroma cacao* (L.) enhance tolerance to *Phytophthora* (Mejía et al., 2008). The endophytic bacteria produce several antimicrobial compounds that can increase the resistance of the plants to various phytopathogenic fungi, bacteria, nematodes, etc. Endophytic *Pseudomonas putida* BP25 associated with black pepper inhibits a range of phytopathogens, viz., *Rhizoctonia solani, Phytophthora capsici, Gibberella moniliformis, Pythium myriotylum, Radopholus similis*, and *Colletotrichum gloeosporioides* by the production of several compounds (Sheoran et al., 2015). *Macrophomina phaseolina* causes charcoal rot disease in different crops and has been reported to be restricted by siderophore-producing *Rhizobium* (Arora et al., 2001). An endophyte, *Pseudomonas fluorescens*, having antagonistic effects against *Vertcillium* was isolated by Mercado-Blanco et al. (2004) from olive plant roots. Yong et al. (1994) established the role of endophyte *Fusarium* spp. in the enhancement of growth and terpenoid content in *Euphorbia pekinensis* Rupr.

The endophytic fungus *Phomopsis cassia* associated with *Cassia spectabilis* enhances tolerance against phytopathogenic fungi *Cadosporium sphaerospermum* and *C. cladosporioides* by producing cadinane sesquiterpenoids, which are toxic to pathogens (Silva et al., 2006). Similarly, Wang et al. (2012) reported that an endophyte, *Chaetomium globosum* L18, inhibits pathogenic fungi by synthesizing some toxic chemicals in *Curcuma wenyujin* Y. H. Chen & C. Ling. In another study, Cao et al. (2009) identified endophytic microbes *Choiromyces aboriginum, Stachybotrys elegans*, and *Cylindrocarpon* associated produces cell wall-degrading enzymes to kill pathogenic fungi in *Phragmites australis* (Cav.) Trin. ex Steud. Furthermore, an endophytic fungus, *Trichothecium roseum*, has a toxic chemical, “trichothecin”, which enhances tolerance to pathogenic fungi in *Maytenus hookeri* Loes. (Zhang et al., 2010). Similarly, *Bacillus subtilis* and *Myxormia* sp. also enhance tolerance against pathogenic fungi *Fusarium oxysporum* and *F. solani* in *Angelica sinensis* (Oliv.) Diels (Yang et al., 2012).

Endophytic microbes *Chaetomium cochliodes, Cladosporium cladosporioides*, and *Trichoderma viride* enhance insect resistance in creeping thistle *via* producing some chemicals toxic to pathogens (Gange et al., 2012). Similarly, Sumarah et al. (2010) reported that fungal endophytes enhance resistance against *Choristoneura fumiferana* insect in red spruce (*Picea rubens* Sarg.). An endophyte, *Leucocoprinus gongylophorus*, increases insect resistance by synthesizing some chemicals antagonistic to ants’ fungal symbiont (Bittleston et al., 2011). Furthermore, the endophyte *Chaetomium* Ch1001 associated with cucumber enhances tolerance against root-knot nematode by producing abscisic acid affecting the motility of the second-stage juveniles of insects (Yan et al., 2011). According to the study undertaken by Gómez-Vidal et al. (2009), endophytes *Beauveria bassiana, Lecanicillium dimorphum*, and *L*. cf. *Psalliotae* increase insect resistance to *Phoenix dactylifera* by modulating the expression of cell division-related proteins in the host plant. Strobel et al. (1999) reported that in thunder god vine (*Triptergyium wilfordii* Hook. f.), an endophye, *Cryptosporiopsis* cf. *quercina*, produces “cryptocin” and “cryptocandin” toxic to pathogenic fungi *Pyricularia oryzae* in host plant. Daungfu et al. (2019) recently identified several endophytes*, Bacillus subtilis LE24*, *Bacillus amyloliquefaciens LE109*, and *Bacillus tequilensis PO80*, isolated from citrus plant having antagonistic effects against pathogens, which might be helpful in the biocontrol of pathogens. All these findings strongly confirm that the presence of endophytes in the host has the potential to increase their tolerance to pathogens through several mechanisms. Conversely, in resistance stimulation to disease facilitation, the process wherein endophytes invade plant tissues affects endophyte–pathogen interactions, perhaps producing facilitation (positive induction of pathogens), negatively strengthening host resistance, or having no effect at all (Suryanarayanan et al., 2009; Schmidt et al., 2014; Adame-Alvarez et al., 2014). These require further research for confirmation.

## Tolerance to abiotic stresses

Abiotic stressful conditions, viz., drought, water-logging, salinity, cold, heat, and heavy metal toxicity, may cause adverse effects on soil and environmental health (Wang et al., 2003; Khare and Arora, 2015). Endophytes have a vital function in increasing tolerance against abiotic perturbations in plants (Wani et al., 2016). Waqas et al. (2012) reported that stomatal closure mediated by abscisic acid (ABA) might have a key function in the regulation of plant growth by reducing other abiotic stresses, including osmotic stress.

Under drought stress, an endophytic bacterium, *Sinorhizobium meliloti*, upregulated FeSOD and CU/ZnSOD, promoting drought tolerance in *Medicago sativa* (L.) (Naya et al., 2007). Similarly, *Arbuseular mycorrhiza* improves nutrient absorption and modifies Salvia’s metabolic activities to boost drought tolerance (Meng and He, 2011). An endophytic microbe, *Trichoderma hamatum DIS 219b*, delayed drought-induced alterations in stomatal conductance and net photosynthesis, promoting drought tolerance in *Theobroma cacao* (L.) (Bae et al., 2009). According to Sziderics et al. (2007), a fungal endophyte, *Piriformospora indica*, enhances osmotic stress tolerance *via* encoding enzyme ACC oxidase and lipid transfer protein. Wang et al. (2009) reported that *Arbuseular mycorrhiza* and *Penicillium griseofulvum* reduce injury of water stress by increasing the activity of protective enzymes and osmotica contents, thereby enhancing tolerance against salt and drought stress in *Glycyrrhiza uralensis.* Liu et al. (2011) reported that soluble protein concentration and peroxidase activity (POD) are altered by *Chaetomium globosum and Botrytis* sp. Under salt stress in *Chrysanthemum morifolium* (Ramat.) Hemsl. A fungal endophyte, *Yarrowia lipolytica*, promotes salinity stress tolerance in *Euphorbia milii* Des Moul. (Jan et al., 2019).

Moreover, ABA-mediated signaling pathways and their biosynthesis during salinity stress are altered by several microbes in the plant endosphere and may help plant growth. *B. phytofirmans* (PsJN) altered gene expression for a cell surface signaling element, which passes signals to bacteria about the alteration in environmental conditions and consequently improves their metabolism (Sheibani-Tezerji et al., 2015). Fernandez et al. (2012) reported in PsJN bacterized grapevine that stress-induced gene expression in addition to metabolite levels improved over control at a lower temperature by balancing carbohydrate metabolism. Recently, de Zélicourt et al. (2018) reported that *Enterobacter* sp. (SA187), an endophyte, colonizes *Arabidopsis* root and shoot tissues and stimulates tolerance against salt stress by synthesizing KMBA (2-keto-4-methylthiobutyric acid). Márquez et al. (2007) reported that at high soil temperatures, *Curvularia protuberate*, an endophytic fungus, has been linked to *Dichanthelium lanuginosum* for its survival. Wan et al. (2012) reported that endophytes can also reduce the heavy metal-promoted oxidative injury. Earlier studies suggested endophytic bacteria’s function in enhancing tolerance to metal toxicity through diverse mechanisms such as sequestration, intracellular accumulation, and extracellular precipitation or alteration of toxic metal ions to a minimum or non-toxic form (Ma et al., 2016; Mishra et al., 2017). Luo et al. (2012) discovered that a bacterial endophyte, *Bacillus* sp. *SLS18*, reduces heavy metal toxicity *via* root tillers and biomass accumulation in *Solanum nigrum* (L.) and *Phytolacca acinosa* Roxb. Recently, Domka et al. (2019) identified an endophytic fungus, *Mucor* sp., which enhances metal toxicity tolerance in *Arabidopsis arinosa* (L.) Lawalrée. Some examples of endophytes enhancing the tolerance of crop plants against abiotic and biotic stresses are presented in **Table 2**.

**Table 2:** Host medicinal plants with enhanced defense responses conferred by endophytic microorganisms.

Endophytic microorganisms	Host plant	Type of stresses	Mechanism	References
*Bacillus subtilis, Myxormia* sp.	*Angelica sinensis* (Oliv.) Diels	Pathogenic fungi: Fusarium oxysporum and F. solani	Produce some chemicals toxic to pathogens	Yang et al., 2012
*Gilmaniella* sp. AL12.	*Atractylodes lancea* (Thunb.) DC.	Pathogenic fungi	Produce jasmonic acid inducing defense responses	Ren and Dai, 2012
*P. indica*	*Capsicum annum* (L.)	Osmotic stress	Encodes enzyme ACC oxidase, encodes a lipid transfer protein	Sziderics et al., 2007
*Phomopsis cassia*	*Cassia spectabilis* DC.	Pathogenic fungi: Cadosporium sphaerospermum, C. cladosporioides	Produce cadinane sesquiterpenoids toxic to pathogens	Silva et al., 2006
*Chaetomium globosum, Botrytis* sp.	*Chrysanthemum morifolium* (Ramat.) Hemsl.	Salt stress	Increase POD activity and soluble protein content	Liu et al., 2011
*Chaetomium cochliodes, Cladosporium cladosporioides, Trichoderma viride*	*Cirsium arvense* (L.) Scop.	Insect	Produce some chemicals toxic to pathogens	Gange et al., 2012
*Leucocoprinus gongylophorus*	*Cordia alliodora Cham.*	Insect	Produce some chemicals antagonistic to ants’ fungal symbiont	Bittleston et al., 2011
*Chaetomium Ch1001*	*Cucumis sativus* (L.)	Insect: root-knot nematode Meloidogyne incognita	Produced abscisic acid affecting motility of the second stage juveniles of insects	Yan et al., 2011
*Chaetomium globosum* L18	*Curcuma wenyujin* Y.H. Chen & C. Ling	Pathogenic fungi	Produce some chemicals toxic to pathogens	Wang et al., 2012
*Arbuseular mycorrhiza, Penicillium griseofulvum*	*Glycyrrhiza uralensis* Fisch. ex DC.	Drought and salt stress	Reduce injury of water stress by increasing protective enzymes’ activity and osmotica contents	Wang et al., 2009
*Trichothecium roseum*	*Maytenus hookeri* Loes.	Pathogenic fungi	Produce trichothecin toxic to pathogens	Zhang et al., 2010
*Sinorhizobium meliloti*	*Medicago sativa* (L.)	Drought stress	FeSOD and CU/ZnSOD are upregulated	Naya et al., 2007
*Pseudomonas koreensis* AGB-1	*Miscanthus sinensis* Andersson	Heavy metal toxicity (Zn, Cd, As, and Pb)	Through extracellular sequestration, increased catalase and SOD activities in plants	Babu et al., 2015
*Beauveria bassiana, Lecanicillium dimorphum*, L. cf. *Psalliotae*	*Phoenix dactylifera* (L.)	Insect	Modulate the expression of cell division-related proteins in host	Gómez-Vidal et al., 2009
*Choiromyces aboriginum, Stachybotrys elegans, Cylindrocarpon*	*Phragmites australis* (Cav.) Steud.	Pathogenic fungi	Produce cell wall-degrading enzymes to kill pathogenic fungi	Cao et al., 2009
150 foliar fungal endophytes	*Picea rubens* Sarg.	Insects: Choristoneura fumiferana	Produce some chemicals toxic to insects	Sumarah et al., 2010
*Arbuseular mycorrhiza*	*Salvia miltiorrhiza* Bunge	Drought stress	Increase the absorption of nutrient and alter metabolic activities in host	Meng and He, 2011
*Bacillus* sp. SLS18	*Solanum nigrum* (L.), Phytolacca acinosa Roxb.	Heavy metal toxicity (Mn and Cd)	Accumulation of root tillers and biomass	Luo et al., 2012
*Trichoderma hamatum* DIS 219b	*Theobroma cacao* (L.)	Drought stress	Delayed drought-induced changes in stomatal conductance and net photosynthesis	Bae et al., 2009
*Cryptosporiopsis* cf. *quercina*	*Triptergyium wilfordii* Hook.f.	Pathogenic fungi: Pyricularia oryzae	Produce cryptocin and cryptocandin toxic to pathogens	Strobel et al., 1999

## Endophytic microorganisms producing secondary metabolites/bioactive compounds in the host plant

The diversity of bioactive compounds varies according to the habitats of the host plants; for example, in a tropical rain forest, limited resource availability leads to great competition among plants and their endophytes; thus, selection pressure is at the peak, resulting in the production of many novel molecules as compared to temperate forests (Reddell and Gordon, 2000). Isolation of indigenous microbes from different plant parts and their interaction with the host plant may divulge applicant microbes to promote plant growth and as biocontrol agents. Such microbial inoculants have the potential to provide resistance against environmental perturbations without affecting the indigenous microbial equilibrium (Palaniyandi et al., 2013).

Many endophytic microbes can synthesize a notable variety of secondary metabolites like antioxidant, anticancer, immunosuppressive, antidiabetic, antioomycete, antifungal, antibacterial, antiviral, and nematicidal agents (Gunatilaka, 2006; Zhang et al., 2006b; Verma et al., 2009; Aly et al., 2011). Shimizu (2011) reported that endophytic actinobacteria synthesize antibiotics, which is useful in plant growth promotion and improves the tolerance of plants to stress. Endophytes also have been in the limelight during the last decade or so because of their capability in producing several secondary metabolites that are bioactive (Tan and Zou, 2001; Schulz et al., 2002; Strobel, 2003; Prado et al., 2013). These compounds extracted from endophytes belong to the various chemical groups like xanthones, terpenoids, phenols, steroids, benzopyranones, isocoumarins, chinones, cytochalasins, tetralones, and enniatines (Schulz et al., 2002). Sometimes, these endophytic microbes may cause variation in well-known structural compounds like fungal steroids, ergosterol, or plant hormone indole-3-acetic acid (Lu et al., 2000). Several endophytes can also be attributed to providing protection of plants against pests because of the compounds occurring in them (Poling et al., 2008).

There are many reports that have shown that host secondary metabolism can be induced by endophytes, but such interaction has not been much explored. Qawasmeh et al. (2012) reported that when endophytes (from the Clavicipitaceae family) interact with grasses, there is a production of phenolic compounds, which are mainly defense-related. However, secondary metabolites could remain unchanged or get reduced depending on which type of endophyte interacts with the host plant. A well-known example of a high-demanding anticancerous molecule is “taxol” isolated from *Taxomyces andreanae*, a taxol-producing fungal endophyte of *Taxus* species (Stierle et al., 1995). An example showed that bacterial endophytes have methanol dehydrogenase genes, which were known to express furanone biosynthesis and localized especially in vascular tissues of strawberry receptacles and plant achenes cells (Nasopoulou et al., 2014). Likewise, Koskimaki et al. (2009) have reported that fungal endophyte *Paraphaeosphaeria* sp. increased the accumulation and biosynthesis of flavan-3-ols phenolic acids and oligomeric proanthocyanidins in *Vaccinium myrtillus*. In *Artemisia annua* (L.), an endophytic bacterium, *Pseudonocardia* sp., has been reported to increase artemisinin synthesis by upregulating the cytochrome P450 monooxygenase (CYP71AVI) and cytochrome P450 oxidoreductase (CPR) genes, and this also activated a defense mechanism (Wang et al., 2006). It has also been found that endophytes may act as upregulators of the specific gene expression for tissue-specific roles, **e.g.,** foliar endophytes enhanced primary metabolites, crop yields could be increased by root endophytes, and endophytes isolated from the capsule could upregulate key genes of benzylisoquinoline alkaloid (BIA) biosynthesis in *Papaver somniferum* (L.) (Pandey et al., 2016a). Tiwari et al. (2010) found an improved content and yield of essential oils in holy basil. In the Chinese medicinal plant *Atractylodes lancea* (Thunb.) DC., the endophytic bacterium *P. fluorescens* could enhance the generation of ROS (reactive oxygen species), which resulted in an increase of sesquiterpenoids (Zhou et al., 2015). Endophytic bacteria *Aranicola proteolyticus, Bacillus cereus, B. thuringiensis, B. licheniformi*, and *Serratia liquefaciens* recovered from *Pinellia ternata* (Thunb.) Makino could produce inosine and guanosine alkaloids similar to their host plant in fermentation media (Liu et al., 2015). Tiwari et al. (2013) found that endophyte *Micrococcus* sp. and *Staphylococcus sciuri* inoculated plant had significantly higher amounts of ajmalicine, serpentine, and vindoline in *Catharanthus roseus* (L.) G. Don. Kumar et al. (2014) reported that the endophytic bacterium *Azotobactor chroococcum* could enhance the yield of curcumin in rhizomes of turmeric.

Endophytic microflora encompasses a high potential for the synthesis of an ample range of unidentified, undepicted novel secondary metabolites within or without host plants. It is needed to identify which type of mechanism or cryptic genes and what circumstances for the evolution of the genome are involved in the synthesis of novel compounds in endophytes as well as *in-planta*. Some examples of endophytes producing secondary metabolites in host plants are presented in **Table 3**.

**Table 3:** Endophytic microorganisms producing plant secondary metabolites in host plants.

Endophytic microorganisms	Host plant	Plant secondary metabolite	Bioactivity of secondary metabolite	References
**Bacterial endophytes**				
*Jishengella endophytica*	*Xylocarpus granatum* J. Koenig	Perlolyrine	Antiviral effect	Wang et al., 2014
*Streptomyces* sp. *TP-A0569*	*Allium fistulosum* (L.)	Fistupyrone	Protection against pathogenic fungi	Bernardi et al., 2019
*Streptomyces* sp. *TP-A0556*	*Aucuba japonica* Thunb.	Coumarins TPU-0031-A and B	Antibiotic activity against Gram-positive and Gram-negative bacteria	Bernardi et al., 2019
*Streptomyces hygroscopicus TP-A0451*	*Pteridium aquilinum* (L.) Kuhn	Pteridic acids A and B, Pterocidin	Plant growth-promoting properties	Bernardi et al., 2019
*Taxomyces andreanae*	*Taxus brevifolia* Nutt.	Taxol	Anticancer	Strobel et al., 1993
*Streptomyces griseus*	*Kandelia candel* (L.) Druce	p-Aminoacetophenonic acids	Antimicrobial	Guan et al., 2005
*Streptomyces NRRL 30562*	*Kennedia nigricans* Lindl.	Munumbicins Munumbicin D	Antibiotic Antimalarial	Castillo et al., 2002
*Serratia marcescens*	*Rhyncholacis penicillata* Matthiesen	Oocydin A	Antifungal	Strobel et al., 2004
*Pseudomonas fluorescens*	*Atractylodes lancea* (Thunb.)DC.	Increases oxygenous sesquiterpenoid content	Triggers generation of ROS	Zhou et al., 2015
*Azotobacter chroococcum*	*Curcuma longa* (L.)	Curcumin	Anti-inflammatory, anti-tumor, and antioxidant	Kumar et al., 2014
*Stenotrophomonas maltophilia*	*Papaver somniferum* (L.)	Enhance alkaloid and morphine contents	Narcotic analgesics	Limón et al., 2015
*Pseudonocardia* sp.	*Artemisia annua* (L.)	Artemisinin	Antimalarial	Sato and Kumagai, 2013
**Fungal endophytes**				
*Acremonium* sp.*, Shiraia* sp.	*Huperzia serrata* (Thunb.) Trevis.	Huperzine A	Anticholinesterase	Li et al., 2007
*Alternaria* sp.	*Phellodendron amurense* Rupr.	Berberine	Antibiotic	Duan et al., 2009
*Alternaria* sp.	*Sabina vulgaris* Antoine	Podophyllotoxin	Antitumor	Lu et al., 2006
*Aspergillus fumigatus*	*Podocarpus* sp. Pers.	Paclitaxel	Antitumor	Sun et al., 2008
*Aspergillus nidulans, A. oryzae*	*Ginkgo biloba* (L.)	Quercetin	Anti-inflammatory	Qiu et al., 2010
*Blastomyces* sp., *Botrytis* sp.	*Phlegmariurus cryptomerianus*	Huperzine A	Anticholinesterase	Ju et al., 2009
*Botryodiplodia theobroma, Fusarium lateritium, Monochaetia* sp.*, Pestalotia bicilia*	*Taxus baccata* (L.)	Paclitaxel	Antitumor	Venkatachalam et al., 2008
*Cephalosporium corda*	*Fritillaria ussuriensis* (Maxim.)	Sipeimine	Antibechic and anti-ulcer	Yin and Chen, 2008
*Cephalosporium* sp., *Paecilomyces* sp.	*Paris polyphylla* var. *yunnanensis* (Franch.) Hand.-Mazz.	Diosgenin	Antitumor, anti-inflammatory, cardiovascular protection	Cao et al., 2007
*Chaetomium globosum*	*Hypericum perforatum* (L.)	Hypericin	Anti-depressant	Kusari et al., 2008
*Cladosporium cladosporio*	*Taxus media* Rehder	Paclitaxel	Antitumor	Zhang et al., 2009
*Cochliobolus nisikadoi*	*Cinnamomum camphora* chvar. *Borneol*	Borneol	Anti-inflammatory, antioxidant	Chen et al., 2011b
*Colletotrichum gloeosporioides*	*Piper nigrum* (L.)	Piperine	Antimicrobial, antidepressant, anti-inflammatory, and anticancer	Chithra et al., 2014
*Entrophospora infrequens, Neurospora* sp.	*Nothapodytes foetida* (Wight) Sleumer	Camptothecin	Antitumor	Amna et al., 2006; Rehman et al., 2008
*Fusarium oxysporum*	*Juniperus recurva* Buch.-Ham. ex D.Don	Podophyllotoxin	Antitumor	Kour et al., 2008
*Fusarium oxysporum*	*Ginkgo biloba* (L.)	Ginkgolide B	Antishock, anti-inflammatory, and antiallergic	Cui et al., 2012
*Fusarium redolens*	*Fritillaria wabuensis* S.Y. Teng & S.C. Yueh	Peimisine and imperialine-3β-D-glucoside	Get rid of sputum, cough, and antitumor	Pan et al., 2015
*Fusarium solani*	*Apodytes dimidiata* E.Mey. ex Arn.	Camptothecin	Antitumor	Shweta et al., 2010
*Fusarium solani*	*Camptotheca acuminata* Decne	Camptothecin	Antitumor	Kusari et al., 2009
*Fusarium solani*	*Taxus celebica* (Warb.) H.L. Li	Paclitaxel	Antitumor	Chakravarthi et al., 2008
*Fusarium solani, Metarhizium anisopliae, Mucor rouxianus*	*Taxus chinensis* Roxb.	Paclitaxel	Antitumor	Deng et al., 2009; Liu et al., 2009
*Monilia* sp., *Penicillium implication*	*Dysosma veitchii* (Hemsl. & E.H.Wilson) L.K.Fu ex T.S.Ying	Podophyllotoxin	Antitumor	Yang et al., 2003
*Ozonium* sp., *Alternaria alternata, Botrytis* sp., *Ectostroma* sp., *Fusarium mairei, Papulaspora* sp., Tubercularia sp.	*Taxus chinensis* var. *mairei* (Lemee & Levl.) W.C. Cheng & L.K. Fu	Paclitaxel	Antitumor	Zhou et al., 2007; Guo et al., 2009; Wu et al., 2013
*Penicillium chrysogenum*	*Lycopodium serratum* Thunb.	Huperzine A	Anticholinesterase	Zhou et al., 2009
*Penicillium implicatum*	*Diphylleia sinensis* H.L.Li	Podophyllotoxin	Antitumor	Zeng et al., 2004
*Penicillium* sp., *Phialocephala fortinii, Trametes hirsuta, Alternaria neesex*	*Sinopodophyllm hexandrum* (Royle) Ying	Podophyllotoxin	Antitumor	Li, 2007
*Pestalotiopsis microspora, Sporormia minima, Trichothecium* sp.	*Taxus wallachiana* Zucc.	Paclitaxel	Antitumor	Shrestha et al., 2001
*Pestalotiopsis pauciseta*	*Cardiospermum helicacabum* (L.)	Paclitaxel	Antitumor	Gangadevi et al., 2008
*Pestalotiopsis terminaliae*	*Terminalia arjuna* (Roxb. ex DC.) Wight & Arn.	Paclitaxel	Antitumor	Gangadevi and Muthumary, 2009
*Phomopsis* sp.*, Diaporthe* sp.*, Schizophyllum* sp.*, Penicillium* sp.*, Fomitopsis* sp.*, Arthrinium* sp.	*Cinchona ledgeriana* Bern. Moens	Cinchona alkaloids: quinine, quinidine, cinchonidine, cinchonine	Antipyretic and antimalarial, analgesic and anti-inflammatory	Maehara et al., 2012
*Phyllosticta citricarpa* *Phyllosticta spinarum*	*Citrus medica* (L.) *Cupressus* (L.)	Paclitaxel	Antitumor	Kumaran et al., 2008
*Phyllosticta dioscoreae*	*Hibiscus rosa-sinensis* (L.)	Paclitaxel	Antitumor	Kumaran et al., 2009
*Sordariomycete* sp.	*Eucommia ulmoides* Oliv.	Chlorogenic acid	Antimicrobial and antitumor	Chen et al., 2010
*Trichoderma atroviride* D16	*Salvia miltiorrhiza* Bunge	Tanshinone IIA and tanshinone I	Antibacterial and anti-inflammatory	Ming et al., 2011

## Phytoremediation

Phytoremediation is the most efficient and eco-friendly system for restoring natural soil conditions when various environmental pollutants have contaminated it. In the past two decades, endophytes’ usage in the phytoremediation of diverse environmental pollutants has received more attention (Sheng et al., 2008; McGuinness and Dowling, 2009; Weyens et al., 2009; Weyens et al., 2010; Hur et al., 2011; Segura and Ramos, 2013; Wei et al., 2014; Anyasi and Atagana, 2018). Such research has shown the potential of plant–microbe interaction in the restoration of polluted regions and may be helpful in designing efficient environmental pollutant removal systems. One of the reasons could be attributed to the complicated relationship between endophytes and their hosts. In contrast, the other reason has to do with the fact that it is practically impossible to comprehend the mechanisms of existence and situations of endophytes to be able to replicate them. It is impractical to exaggerate the role of endophytes in both management and harnessing the natural environment. Unfortunately, the holistic view of these taxa has been constrained by them. Such constraints include the fact that the process of isolation of endophyte relies on culture dependence, while several microorganisms exist that cannot be cultured. This emphasizes nonculture-dependent innovation as the barrier to understanding endophytes.

## Agronomic practical application of endophytic microorganisms

The most frequently used inoculation technique in agriculture includes the use of endophytes; culture, facilitated by a carrier, is combined with the synthetically manufactured sticky seeds and sown. Moreover, several commercial formulations include liquid cultures directly applied or with the granular fertilizer or seed applications. Other techniques have also been used, including seed priming, seed coating, foliar spraying, root dipping, pelleting, and direct soil application (O’Callaghan, 2016). Given the presence of endophytic microbes, seed inoculation can accomplish this goal. Still, its effectiveness is constrained by the lengthy engaging period, subsequent physical abrasion, and competition with other soil microbes. Root dipping or seedlings treated with microbial suspension are also susceptible to contamination and handling issues. The application of endophytes in bulk populations is made possible by pelleting or direct soil application. However, there is still an issue with the lack of homogeneity in the field and exposure to environmental perturbations. Despite using every conventional method, the microbes still need to endure for a few weeks before they may enter the plant after root hair emergence. Even while some endophytes’ facultative character suggests the prospect of further colonization if they can endure in the rhizosphere, the ecological constraint exists. Seed priming with a predetermined duration can help the microbe’s entrance during imbibitions. However, several scientific and technical difficulties are still associated with applying endophytes to seeds *via* coatings, sprays, granules, and capsules. Moreover, a seemingly simple procedure like plate counting also seems to have some technical problems, e.g., the “viable but not culturable” bacteria may not be detected by plate counting.

The formulation should facilitate the microorganism’s penetration and colonization of the host while minimizing dosage and cost. It should also increase microbe establishment in the soil and close to or on the plant. The application of endophytes requires a thorough knowledge of the physicochemical and biological environment, including the phyllosphere, soil, seed surface, and rhizosphere, as well as the cultivation and formulation of the biologicals to prolong shelf life. In the context of mycorrhiza, progress has been made in understanding the molecular plant–microbe interaction that needs to be integrated into novel formulation and application strategies. In other circumstances, such endophytic entomopathogenic fungi do not clearly understand how they invade and colonize. Basic research studies only use straightforward water-spore mixtures instead of more advanced and practical application methods.

Furthermore, designing formulations having high microbial inoculant concentration and survivability during storage is crucial for developing potent inoculants. Since it is impossible to test out every potential combination of parameters throughout the formulation process, it is challenging to determine the most critical variables. Nevertheless, it is challenging to maintain sterility throughout the formulation process for an extended period, which could lead to contamination. Even though we can find entomopathogenic fungi in nature in this form, their poor recovery rate from plants reveals that these organisms do not occur naturally.

## Conclusion

Plant–microbe interactions benefit the all-embracing vicinity of agricultural applications. Microorganisms are abundantly present in nature and primarily colonized *in-planta.* Plant–endophyte interaction is mostly considered beneficial, having profound effects on the physiology of the host plant and the overall performance by promoting growth, development, and imparting fitness to the host plants against different biotic and abiotic stresses. They play a vital function in agricultural sustainability by providing eco-friendly inputs to enhance crop productivity and quality while minimizing harmful chemical fertilizers. The study of these plant–microbe interactions helps us acknowledge natural events that influence our daily lives and could benefit befalling in sustainable resources, a smaller influence on the atmosphere and surroundings, and control of environmental pollution. The benefits of using these interactions for biotechnological applications are huge. The utilization of the pre-existing plant–microbe interactions for the promotion of growth of the plant and biocontrol diminishes the use of unnatural synthetic pesticides and fertilizers, resulting in lowering input costs and, more importantly, reducing the influence of chemical nutrients and pesticides on existing useful fiora and fauna. Moreover, the production of beneficial compounds of industrial and pharmaceutical importance through plant–microbe symbiosis reduces the requirement to supply expensive catalysts and precursors and is energy-saving. In recent years, MAPs are being paid considerable attention worldwide due to their vast economic potential, primarily in the field of herbal medicine. Until the arrival of advanced medicines, an oversized population in emerging nations has traditionally relied upon the products obtained from plants. Furthermore, about 12.5% of the more than 422,000 plant species have been universally documented for medicinal properties; however, only a couple of hundreds are known to be in cultivation. There is a need to grow MAPs to maintain their steady supply and conservation amidst decreasing stocks from natural sources and rising global interest.

### Limitations and further investigations

Endophytic microorganisms are tissue specific in nature; their establishment and functionality within the host are affected by several factors such as tissue type, host’s genotype, and surrounding conditions. The lack of knowledge about the widespread presence of endophytic microorganisms’ communities in plant tissues has been a hindrance in advancing research on endophytes in various fields. It should be noted that the development of successful endophyte application technologies would fully depend on improving our understanding of how they enter and colonize *in-planta*. Consequently, to guarantee reproducibility, reliable methods of endophytic inoculum delivery should be developed for better productivity of MAPs. Leveraging the relationship between plants and endophytes can be crucial for advancing sustainable development (Ryan et al., 2008); extensive research investigations are required to accept or refute this hypothesis. Therefore, in-depth future studies are needed to demonstrate an improved comprehension of the organism in its host to advance the viability of endophyte-assisted biological applications, especially in the field. The persistent reliance on the deployment of a generic method in their processing was inferred to be a hindrance to the capability to fully grasp the interaction between endophytes and their host concerning their utilization in biological activities. Since most organisms prefer to eschew them due to transformations, they cannot be recognized using those general techniques. Thus, using complex molecular processes in their processing will lead to a better understanding and enable the use of the endophytic application in agriculture/food processing, medicine, and environmental management.

## Author contributions

AK and AT conceived and planned this review article. AT and PP wrote the original draft of the manuscript. ST helped in review and data collection. PP prepared the figures. All authors reviewed and agreed on the final version.

## Acknowledgments

We are thankful to the Director of CSIR-Central Institute of Medicinal and Aromatic Plants for providing the necessary facilities to carry out the research work. AT is grateful to the Department of Science and Technology, India for the INSPIRE fellowship.

## Conflict of interest

The authors declare that the research was conducted in the absence of any commercial or financial relationships that could be construed as a potential conflict of interest.

## Publisher’s note

All claims expressed in this article are solely those of the authors and do not necessarily represent those of their affiliated organizations, or those of the publisher, the editors and the reviewers. Any product that may be evaluated in this article, or claim that may be made by its manufacturer, is not guaranteed or endorsed by the publisher.
